# Lectins identify distinct populations of coelomocytes in *Strongylocentrotus purpuratus*

**DOI:** 10.1371/journal.pone.0187987

**Published:** 2017-11-10

**Authors:** Wen-Yun Liao, Sebastian D. Fugmann

**Affiliations:** 1 Department of Biomedical Sciences, Chang Gung University, Kwei-Shan District, Tao-Yuan, Taiwan; 2 Division of Microbiology, Graduate Institute of Biomedical Sciences, Chang Gung University, Kwei-Shan District, Tao-Yuan, Taiwan; 3 Chang Gung Immunology Consortium, Chang Gung Memorial Hospital and Chang Gung University, Kwei-Shan District, Tao-Yuan, Taiwan; 4 Department of General Surgery, Chang Gung Memorial Hospital, Tao-Yuan, Taiwan; University of Liverpool, UNITED KINGDOM

## Abstract

Coelomocytes represent the immune cells of echinoderms, but detailed knowledge about their roles during immune responses is very limited. One major challenge for studying coelomocyte biology is the lack of reagents to identify and purify distinct populations defined by objective molecular markers rather than by morphology-based classifications that are subjective at times. Glycosylation patterns are known to differ significantly between cell types in vertebrates, and furthermore they can vary depending on the developmental stage and activation states within a given lineage. Thus fluorescently labeled lectins that recognize distinct glycan structures on cell surface proteins are routinely used to identify discrete cell populations in the vertebrate immune system. Here we now employed a panel of fifteen fluorescently-labeled lectins to determine differences in the glycosylation features on the surface of *Strongylocentrotus purpuratus* coelomocytes by fluorescence microscopy and flow cytometry. Eight of the lectins (succinylated wheat germ agglutinin, *Len culinaris* lectin, *Pisum sativum* agglutinin, *Saphora japonica* agglutinin, *Solanum tuberosum* lectin, *Lycopersicon esculentum* lectin, *Datura stramonium* lectin, *Vicia villosa* lectin) showed distinct binding patterns to fixed and live cells of three major coelomocyte classes: phagocytic cells, red spherule cells, and vibratile cells. Importantly, almost all lectins bound only to a subgroup of cells within each cell type. Lastly, we established fluorescently-labeled lectin-based fluorescence activated cell sorting as a strategy to purify distinct *S*. *purpuratus* coelomocyte (sub-)populations based on molecular markers. We anticipate that this will become a routine approach in future studies focused on dissecting the roles of different coelomocytes in echinoderm immunity.

## Introduction

The immune system of mammals is based on a complex and intricate interplay of large numbers of distinct immune cells types including the B and T lymphocytes that form the cellular basis of adaptive immunity. While a vast body of knowledge has been accumulated about mammalian immunity, far less is known about the properties and roles of different cell types that constitute the potentially almost equally complex defense systems of higher invertebrates like for example *Strongylocentrotus purpuratus* (purple sea urchin), an echinoderm. Although the *S*. *purpuratus* genome sequencing project revealed the existence of hundreds of homologs of vertebrate immune genes, their roles in sea urchin immune responses is only beginning to be uncovered. Coelomocytes are thought to represent the main professional immune cells of sea urchins, and based on their morphological features they can be separated into four distinct types: phagocytes, vibratile cells, red spherule cells, and colorless spherule cells [1]. As indicated by their name, phagocytes have the ability to take up and degrade bacteria [2], but many properties of this cell type and the role of the other coelomocytes during echinoderm immune responses remain elusive.

In depth studies of murine and human immune cells were largely facilitated by the development of reagents and tools to distinguish between and to purify different cell types and subpopulations thereof based on molecular markers. Such purified cells allowed subsequent comprehensive studies of their gene expression profiles and their function. Monoclonal antibodies directed against unique cell surface markers represent the most commonly used reagents for identifying individual immune cell types [3]. Unfortunately, the use of existing monoclonal antibodies for studies outside the mouse and human species is limited to those directed against highly conserved cell surface molecules. Thus their utility is largely restricted closely related species. Many of the monoclonal antibodies specific for mouse and human immune cell markers do not work in the most distant jawed vertebrates, the cartilaginous fish, and even less so for invertebrates including sea urchins. As the generation of monoclonal antibodies against coelomocyte surface proteins is a lengthy and time-consuming process, we decided to focus on potential alternative molecular determinants: post-translational modifications of cell surface proteins that might differ between distinct coelomocyte populations.

Glycosylation is a common post-translational modification present in extracellular domains of receptor membrane molecules of a wide range or organisms [4]. Glycosylation is found in all eukaryotic organisms, and sugar moieties are transferred to what ends up being the extracellular domain of proteins. This is mediated by glycosyltransferases in the lumen of the endoplasmic reticulum and the golgi [5]. A wide range of studies using mouse and human cells revealed that glycosylation patterns can vary between cell types and the differentiation stage and activation state of individual cells [6–9]. Historically, lectins, proteins that bind to distinct glycosylation patterns, had already been utilized to define different immune cell populations prior to the inventions of monoclonal antibody reagents, and for some purposes they are still a standard tool. One prominent example is the identification of activated germinal center B cells to which peanut agglutinin (PNA), a plant lectin, binds strongly [10]. In contrast, small resting splenic B cells express different glycosyltransferases and hence their surface proteins are not recognized by PNA. Furthermore, unique glycosylation patterns and their detection using lectins is an emerging theme in cancer diagnosis [11–13].

Based on these observations on vertebrate cells, we here utilized a commercially available panel of fluorescently labeled plant lectins to screen for distinct binding patterns of these molecules to *S*. *purpuratus* coelomocytes. We identified several lectins that bind distinct coelomocyte types and/or subpopulations thereof. Furthermore, we demonstrate their suitability for fluorescence microscopy and flow cytometry experiments to separate and purify sea urchin coelomocytes.

## Material and methods

### Lectins and competitors

All fluorescently-labeled lectins and the competitors for the lectin binding assay (chitin hydrolysate, α-methylmannoside, and N-acetylgalactosamine) were obtained from a commercial vendor (Vector Laboratories) (Table 1).

**Table 1:** Fluorescence dye- coupled lectins for cell surface staining of *S*. *purpuratus* coelomocytes.

Name	Description	Main specificities^a^	Fluorophore
sWGA	Wheat Germ agglutinin, succinylated	oligosaccharides containing terminal N-acetylglucosamine or chitobiose; sialic acid residues are not bound	rhodamine
LCA	*Len culinaris* lectin	α-linked mannose residues; an α-linked fucose residue attached to the N-acetylchitobiose of the core oligosaccharide significantly enhances affinity	rhodamine
PSA	*Pisum sativum* agglutinin	α-linked mannose-containing oligosaccharides, with an N-acetylchitobiose-linked α-fucose residue included in the receptor sequence; calcium and manganese ions are required for binding	rhodamine
GSL-1	*Griffonia simplicifolia* lectin I	Galactose	rhodamine
PHA-L	*Phaesolus vulgaris* leucoagglutinin	complex structures	rhodamine
PHA-E	*Phaesolus vulgaris* erythroagglutinin	complex structures	rhodamine
SJA	*Saphora japonica* agglutinin	carbohydrate structures terminating in N-acetylgalactosamine and galactose residues, with preferential binding to β anomers	rhodamine
PNA	Peanut agglutinin	Galactose	fluorescein
ECL	*Erythrina cristagalli* lectin	Galactose	fluorescein
Jacalin	Jacalin	Galactose	fluorescein
STL	*Solanum tuberosum* lectin	oligomers of N-acetylglucosamine and some bacterial cell wall oligosaccharides containing N-acetylglucosamine and N-acetylmuramic acid; similar but not identical specificities as WGA and DSL	fluorescein
LEL	*Lycopersicon esculentum* lectin	shares some specificities with STL, DSL, and WGA, but has been reported to be dissimilar in many respects; binds to glycophorin and Tamm-Horsfall glycoprotein and labels the vascular endothelium in rodents	fluorescein
DSL	*Datura stramonium* lectin	(β-1,4) linked N-acetylglucosamine oligomers, preferring chitobiose or chitotriose over a single N-acetylglucosamine residue	fluorescein
GSL-2	*Griffonia simplicifolia* lectin II	N-acetylglucosamine	fluorescein
VVL	*Vicia villosa* lectin	α- or β-linked terminal N-acetylgalactosamine, in particular a single α-N-acetylgalactosamine residue linked to serine or threonine in a polypeptide (the Tn antigen)	fluorescein

^a^The information about the binding specificities was obtained from Vector Laboratories (https://vectorlabs.com).

### Animals

*S*. *purpuratus* were originally collected of the California coast, and maintained in sea water at the Academia Sinica ICOB Marine Research Station in Jiaoxi, Taiwan. We obtained all sea urchins from there (a kind gift from Dr. Yi-Hsien Su, Academia Sinica, Taiwan) and maintained them in artificial sea water (Reef Salt, AZOO) at 14°C. All individuals used for the experiments were apparently healthy, and were kept in our facility for at least 3 month prior to the experiments to minimize influences from environmental changes.

### Coelomocyte preparations

Coelomic fluid was drawn from individual sea urchins using a 1 ml syringe with a 25G×1” gauge needle through the peristomial membrane. The syringe was preloaded with an equal volume of ice-cold calcium and magnesium free sea water containing EDTA and imidazole (CMFSW-EI, 0.53 M NaCl, 0.01 M KCl, 0.0024M NaHCO_3_, 0.011 M Na_2_SO_4_, 0.03 M EDTA, 0.05 M imidazole) to prevent clotting. Coelomocyte numbers were determined using a hemocytometer.

To obtain coelomocyte preparations that are highly enriched for phagocytic cells, a mix a vibratile and colorless spherule cells, and red spherule cells we used a density gradient centrifugation procedure established by Gross et al. [14]. Briefly, 5 ml of coelomic fluid was overlaid on a step gradient of 70%, 30%, 20%, 10% and 5% Optiprep (Sigma) in CMFSW-EI and spun at 1500×g at 4°C for 30 min. Individual cell fractions were retrieved from the density gradient using pasteur pipets and diluted into 12 ml of CMFSW-EI. Cell were pelleted at 450×g at 4°C for 5 min, and resuspended in CMFSW-EI. The identity and purity of the cell preparations were determined using phase contrast microscopy.

### Fluorescence microscopy

For observing lectin binding patterns on fixed cells, 100 μl aliquots of coelomocyte suspensions were added to poly-L-lysine coated glass slides (Thermo Scientific Superfrost Plus), and allowed to settle down for 30 min at room temperature in a humidified chamber. The slides were washed with 2 ml calcium and magnesium free sea water (CMFSW, 0.53 M NaCl, 0.01 M KCl, 0.0024M NaHCO_3_, 0.011 M Na_2_SO_4_) to remove non-adherent cells, 100 μls of 4% paraformaldehyde in CMFSW were added, and each sample was fixed for 10 min at room temperature. The excess fixation solution was removed by washing with 3 ml phosphate buffered saline (PBS, 10 mM Na_2_HPO_4_, 1.8 mM KH_2_PO_4_, 137 mM NaCl, 2.7 mM KCl), 100 μl of blocking solution (PBS containing 5% bovine serum albumin, BSA) were added, and incubated for 30 min at room temperature. After washing with 3 ml of PBS, 50 μl microliter of staining solution (PBS containing 1% BSA and 0.5 μg of fluorescein-conjugated lectin or 1 μg of rhodamine-conjugated lectin) were added, and incubated for 1 h at room temperature. Lastly, the slides were washed once with 3 ml PBS, stained with 50 μl 2-(4-Amidinophenyl)-6-indolecarbamidine (DAPI) solution (300 nM in PBS), washed withed 3 ml PBS, mounted with 8 μl 1, 4-Diazobicyclo-(2,2,2-octane) mounting medium (2.5% DABCO in 10 mM Tris pH = 7.5 containing 70% glycerol), and coverslips were sealed with nail polish. For the competition assays, α-methylmannoside or N-acetylgalactosamine were added at the staining step to a final concentration of 0.2 M, or chitin hydrolysate solution was added to make up 20% of the final volume.

For the observation of lectin binding to live coelomocytes, 10 μl of a coelomocyte suspension were added to coated microscope slides (Thermo Scientific Superfrost Plus) and allowed to settle down for 15 min at room temperature in a humidified chamber. After washing the slides with 1 ml of CMFSW-EI, 50 μl of staining solution containing (1 μg of fluorescein-conjugated lectin or 2 μg of rhodamine-conjugated lectin, respectively) was added, and the samples were incubated at room temperature in a dark humidified chamber for 30 min. Subsequently, cells were washed once with 1 ml of CMFSW-EI, covered with a coverslip, and immediately observed by microscopy without sealing.

To stain coelomocytes in suspension, cells were pelleted by centrifugation at 434×g and the supernatant was discarded to remove glycosylated proteins present in the coelomic fluid. Subsequently, the coelomocytes were resuspended in the same volume of CMFSW-EI, and separated into aliquots of 100–200 μl in volume. Then individual fluorescently labeled lectins were added (8 μg/ml WGA-rhodamine, 40 μg/ml LCA-rhodamine, 40 μg/ml PSA-rhodamine, 6.6 μg/ml GSL I rhodamine, 6.6 μg/ml PHA-L-rhodamine, 6.6 μg/ml PHA-E-rhodamine, 6.6 μg/ml SJA-rhodamine, 3.3 μg/ml PNA-fluorescein, 3.3 μg/ml ECL-fluorescein, 3.3 μg/ml Jacalin-fluorescein, 8 μg/ml STL-fluorescein, 8 μg/ml LEL-fluorescein, 6.7 μg/ml DSL-fluorescein, 3 μg/ml GSL II-fluorescein, 6.7 μg/ml VVL-fluorescein,–final concentrations) and incubated for 30 min on ice in the dark. To remove the staining solution, cells were spun down again at 434×g for 5 min at 4°C, resuspended in the same volume of CMFSW-EI, and 10 μl of the suspension was added to uncoated microscopy slides. Lastly, the cells were covered coverslips, and immediately observed by microscopy without sealing.

All slides were observed using a Zeiss Axioimager.Z2 microscope with an Apotome.2 structured illumination accessory. Color images were taken using a digital SLR camera (Canon) attached to an optical port, and fluorescence images were taken using a cooled CCD camera. Raw image files were processed using the Zeiss AxioVision software.

### Analytical flow cytometry

To remove glycosylated proteins present in the coelomic fluid, coelomocytes were spun down at 434×g for 5 min at 4°C, resuspended in the same volume of CMFSW-EI, and separated into 100 μl aliquots. Individual fluorescence-labeled lectins (1 μg sWGA-rhodamine, 2 μg LCA-rhodamine, 2 μg PSA-rhodamine, 0.3 μg STL-fluorescein, 0.3 μg LEL-fluorescein, 0.3 μg DSL, or 1 μg VVL-fluorescein) or pairs thereof were added, and cells were incubated for 30 min on ice in the dark. Stained cells were washed once with 500 μl CMFSW-EI, resuspended in 300 μl CMFSW-EI and subjected to flow cytometry on a FACScalibur flow cytometer (Becton Dickinson) using PBS as the sheath fluid. For the competition assays, α-methylmannoside or N-acetylgalactosamine were added at the staining step to a final concentration of 0.2 M, or chitin hydrolysate solution was added to make up 20% of the final volume.

### Cell sorting

To sort coelomocytes, 1 ml of a 1:1 mix of coelomic fluid and CMFSW-EI were pelleted, resuspended in 1.5 ml and stained with LCA-rhodamine and DSL-fluorescein (40 μg/ml and 6.7 μg/ml final concentrations, respectively) for 30 min on ice in the dark. Subsequently, the cells were washed with 2.5 ml of CMFSW-EI, and pelleted by centrifugation with 434×g for 5 min at 4°C. After resuspending the cells in 1 ml of CMFSW-EI, individual populations were sorted based on their fluorescence profile into 500 μl EasyPure Total RNA reagent (BIOMAN) using a FACS Aria II flow cytometer in the Core Instrument Center of Chang Gung University College of Medicine.

### Gene expression analysis

RNA was purified using EasyPure Total reagent (BIOMAN) using 10 μg of glycogen (Roche) as a carrier according to the manufacturer’s protocol. Approximately 150 ng of RNA were converted into cDNA using the ToolsQuant II Fast RT Kit (BIOTOOLS) kit with random hexamer primers according to the manufacturer’s instructions.

Gene expression levels were quantified by qPCR using the FAST SYBR green Master Mix (Applied Biosystems) and gene specific primers (Table 2) in an ABI 7500 Fast Real-Time PCR machine (Applied Biosystems). Ten microliter reactions were set up containing 5 μl SYBR green Master Mix, 0.2 μl forward primer (10μM), 0.2 μl reverse primer (10μM), 3.6 μl water, and 1 μl cDNA template, and subjected to the following PCR program: 20 sec at 95°C, followed by 40 cycles of 3 sec at 95°C and 30 sec at 60°C. Transcript levels were calculated using standard curves, and normalized to the expression level of a house keeping gene (*Sp-Rpl39*) in each sample.

**Table 2:** Oligonucleotides to test gene expression.

Gene	Name	Nucleotide sequences (5’–3’)	Cell fraction^a^
*Sp-Rpl39*	SPU_18884QF	AGGACAGGCAACAGGATTCG	all
	SPU_18884QR	TGAGTCTGGGCTGATGCTTG	
*Sp-Egr*	WHL22.280477F	CATCGCCGCACCACAATATG	ph^b^
	WHL22.280477R	GGATAACTGCTGGGTCCTCG	
*Sp-FoxJ1*	WHL22.468365F	TCAAGAAGAGACGACACGCC	vcs^c^
	WHL22.468365R	TGTGGTGCTTGGTATCGTCC	
*Sp-Giant*	WHL22.668554F	CATACACCGAGCCGACACTG	rs^d^
	WHL22.668554R	TTTCAGCGACGGGGTAGTTC	
*Sp-B7L3*	SPU20457QF	TTCGGCTGCCTAACGAAACT	ph
	SPU20457QR	CACCAGGAGAAACACGACGA	
*Sp-P2rx4*	SPU19489F	GTTAAACTTCGCATCGGGCG	vcs
	SPU19489R	TGTCTTCTGCGGTCCATTCTC	
*Sp-Pks1*	SPU_002895Q2F	GCAAGCAGTTCAAGGCTCAG	rs
	SPU_002895Q2R	TTGTCTTCCCATGTTGCCGT	

^a^fractions were obtained by density gradient fractionation using a step gradient

^b^phagocytic cells

^c^mix of vibratile and colorless spherule cells

^d^red spherule cells

## Results

### Fluorescence microscopy of fixed coelomocytes

**Fig 1:**
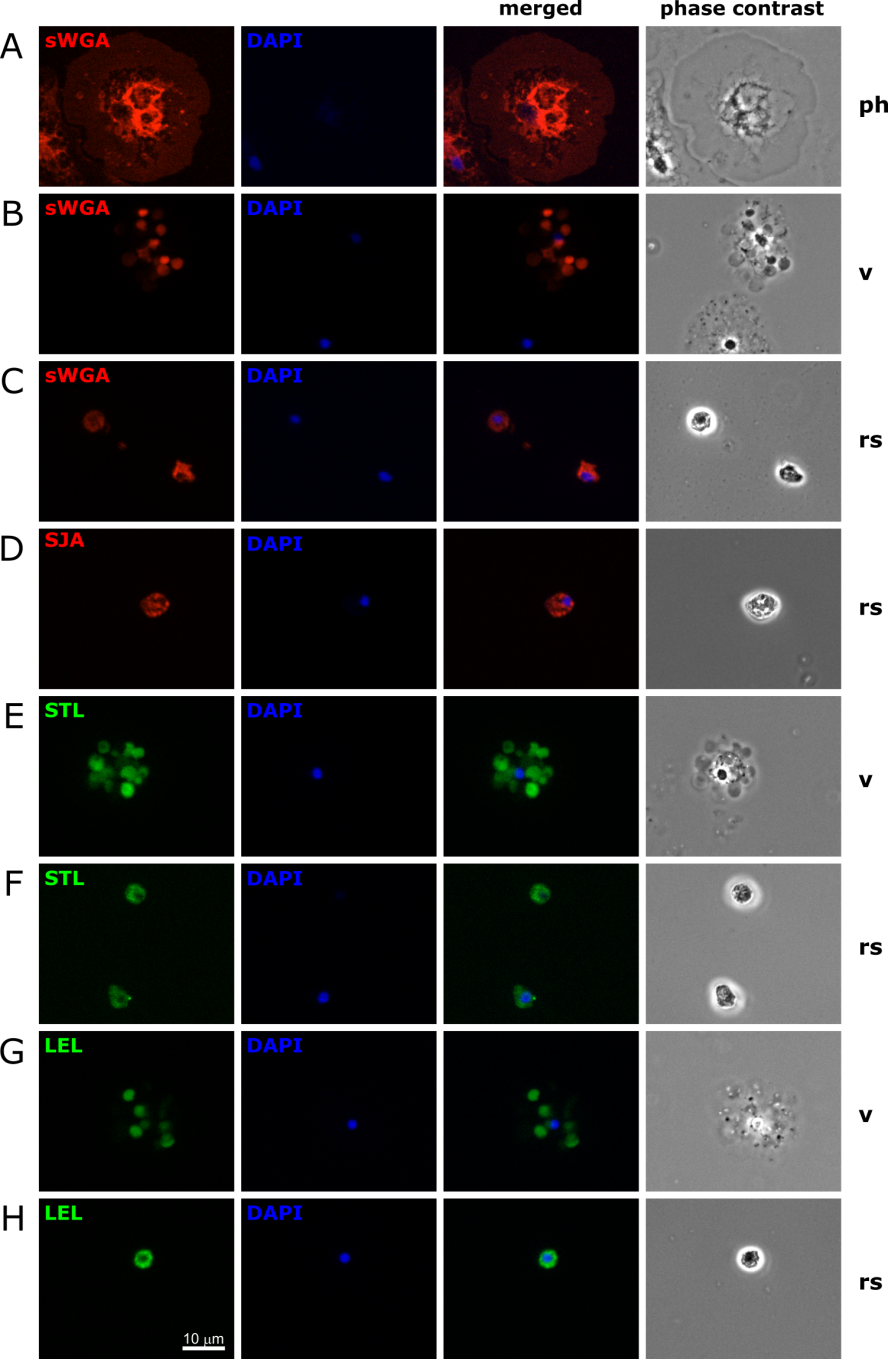
Lectin staining of fixed coelomocytes. Total coelomocytes were separated over a density gradient to obtain cell fractions enriched for phagocytes (ph), vibratile cells (v), and red spherule cells (rs). Cells were settled on glass slides, fixed with paraformaldehyde, and stained with DAPI and the indicated lectins that were labeled with (**A**-**D**) rhodamine or (**E-H**) fluorescein. Note that only staining patterns consistent across three individual are shown. Representative images were taken on a Zeiss Axioimager.Z2 microscope with an Apotome.2 structured illumination accessory using a Plan-Apochromat 40x objective and a cooled CCD camera. Respective phase contrast images were taken (without the Apotome.2 feature) to confirm the identity of each cell. The images for the fluorescent channels are shown individually and merged.

**Fig 2:**
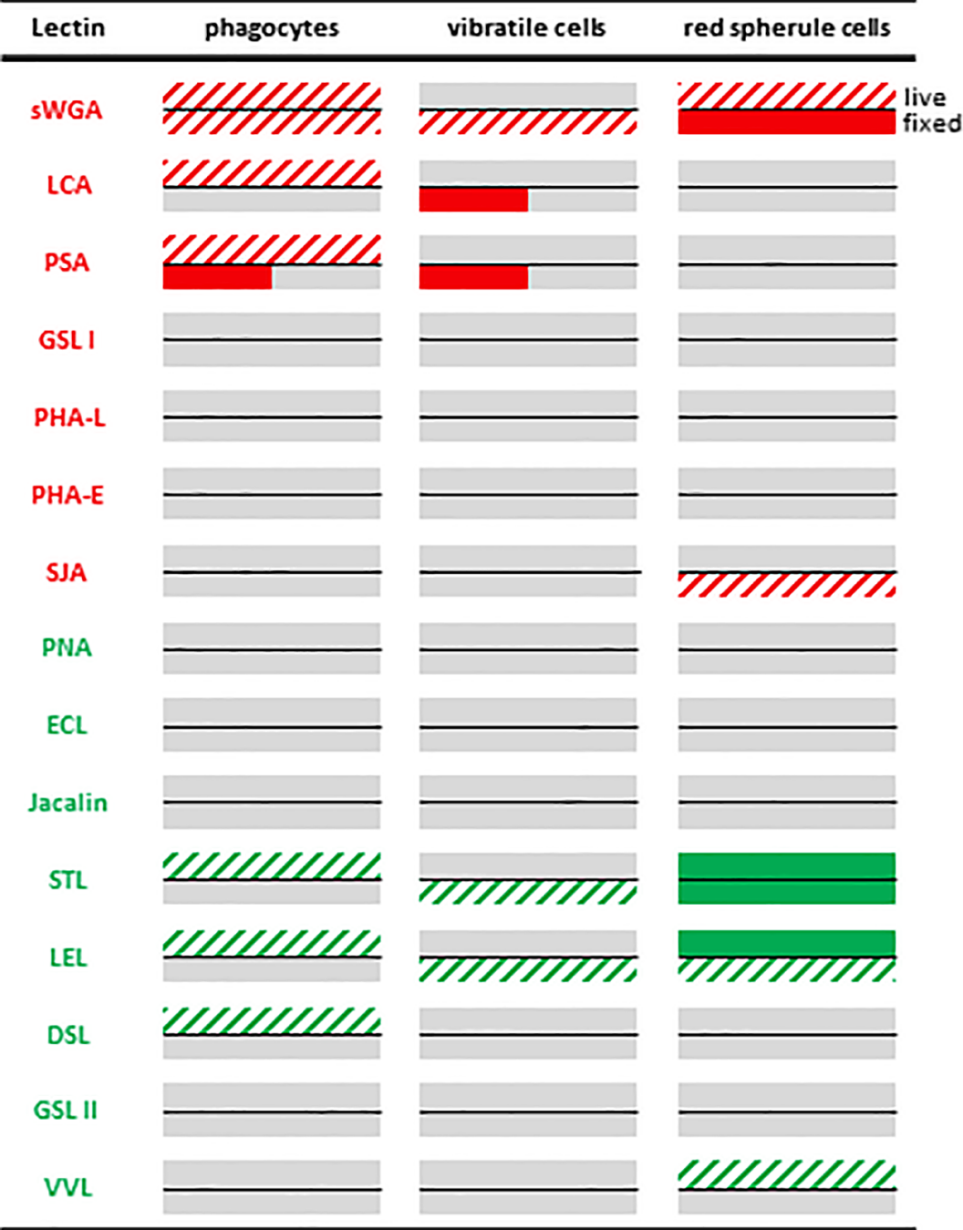
Overview of the lectin binding profiles of live and fixed coelomocytes using fluorescence microscopy. The staining profile for each lectin for each cell type obtained by analyzing at least three individual *S*. *purpuratus* is shown. For each lectin/cell type pair the upper half of the rectangle corresponds to live cell conditions and the lower half the fixed cell data. Solid bars indicate that all cells of a given type were either bound by the lectin (red or green) or not (grey). Diagonal red or green stripes indicate that only some but not all cells were stained. The small solid bars for PSA and LCA for fixed cells indicated that the respective lectin stained this cell type only in some but not all of the sea urchins we tested (for representative images see S1 Fig).

There are currently few molecular markers available to identify distinct coelomocyte populations in *S*. *purpuratus*. This includes the SpTrf (previously refered to as Sp185/333) protein family that is expressed in is distinct types of phagocytic cells but not in spherule or vibratile cells [15,16], complement receptors expressed on polygonal phagocytes [17], and Toll-like receptors (TLRs) expressed largely on phagocytic cells [18]. Lectins bind to distinct glycosylation patterns and fluorescently-labeled lectins are frequently used in combination with monoclonal antibodies to identify specific vertebrate immune cell populations, e.g. PNA binds to glycosylated surface proteins on activated germinal center B cells but not to respective moieties on resting splenic B cells [10]. To screen whether different *S*. *purpuratus* coelomocytes carry distinct glycosylation patterns that can be recognized by different lectins, we selected a test panel of fifteen commercially available lectins that were covalently coupled to either fluorescein or rhodamine, respectively (Table 1). First, coelomic fluid samples were drawn from individual adult sea urchins, and aliquots of these mixed coelomocyte populations were settled on poly-L-lysine coated glass slides. After fixation with paraformaldehyde, the cells were stained with a different fluorescently labeled lectin and DAPI to visualize the nuclei. The labeling patterns of each lectin on individual coelomocytes were observed by fluorescence microscopy and the type of each coelomocyte was identified by phase contrast microscopy. Six of the fifteen lectins showed readily observable fluorescence signals as compared to unstained samples for some coelomocytes: sWGA, LCA, PSA, SJA, STL, and LEL (Fig 1, S1 Fig, S2A, S2B and S2C Fig and data not shown). In contrast, the remaining nine lectins (GSL-1, GSL-2, PHA-L, PHA-E, PNA, ECL, Jacalin, DSL, and VVL) did not show any evidence of binding to any of at least 500 individual coelomocytes observed. While phagocytic cells make up the majority of the coelomocytes (70–85%), vibratile cells (5–15%), red spherule cells (5–15%), and in particular colorless spherule cells (1–2%) typically make up a much smaller proportion [2]. Thus a robust comprehensive assessment of the staining patterns of these less frequent coelomocyte types is difficult to obtain from crude mixed population samples. In addition, some of morphological features of the latter three cell types are less clear after fixation, making it even harder to scan an appropriate number of cells classified unequivocally based on their morphology. Therefore coelomocytes were purified by density gradient centrifugation which yields three fractions: phagocytic cells, red spherule cells, and a mix of vibratile and colorless spherule cells; the lectin binding experiments were repeated with these samples (Fig 1). While the staining patterns of phagocytic cells, red spherule cells, and vibratile cells could be readily assessed using this approach (Fig 2), the number of colorless spherule cells was still too low to discern any clear pattern. Importantly no differences were observed between the experiments with mixed coelomocyte population and those with purified populations. Lastly, to test the specificity of the lectin binding, competition assays were performed using chitin hydrolysate (for sWGA, LEL, and STL) and N-acetylgalactosamine (for SJA). These commercially available substances readily inhibited binding of the corresponding lectins to coelomcytes in all cases (S3 Fig).

**Fig 3:**
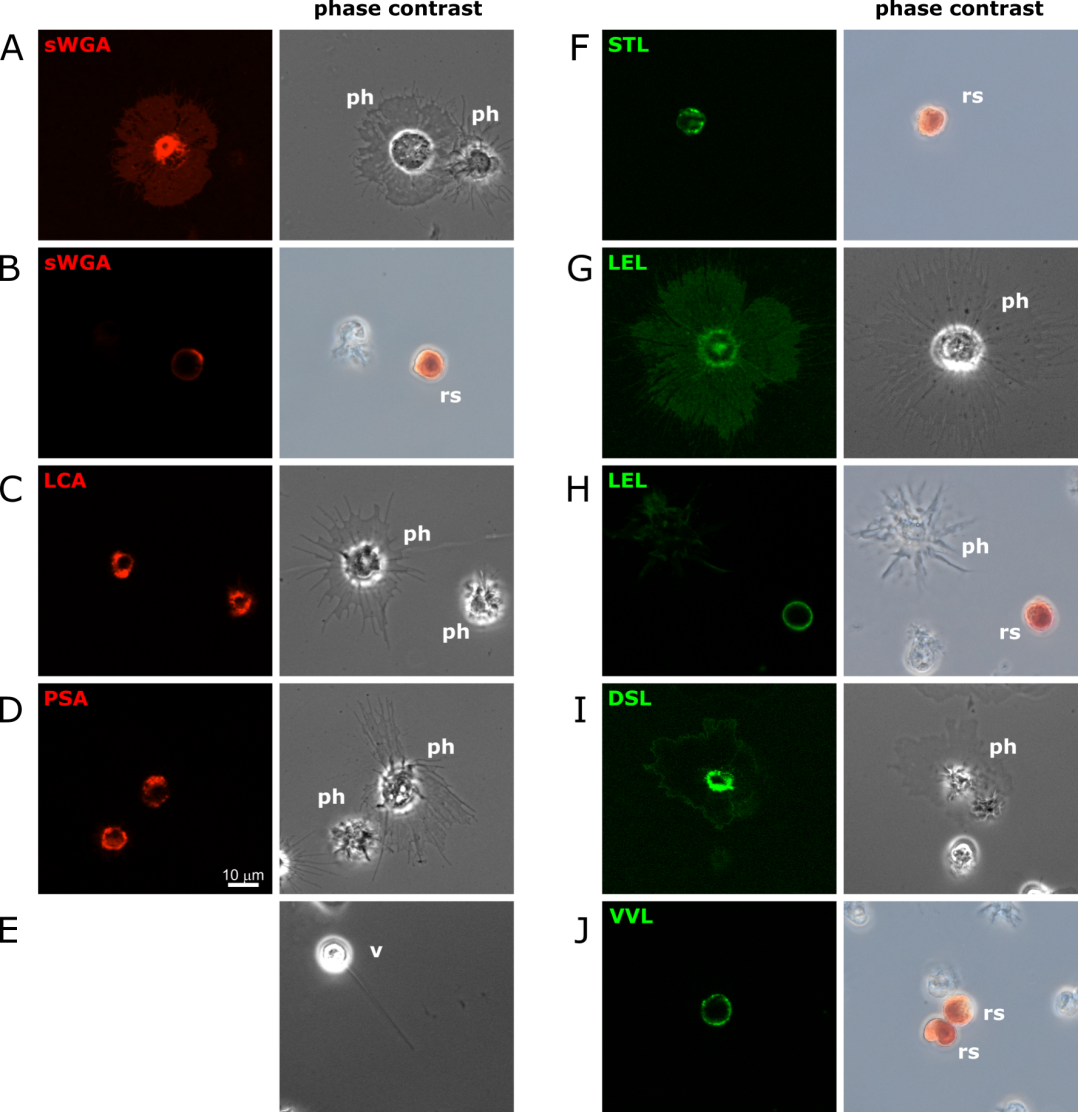
Lectin staining of live settled coelomocytes. Total coelomocytes were stained with the indicated fluorescently labeled lectins (**A**-**D**: rhodamine; **F**-**J**: fluorescein), and then settled onto glass slides. Representative fluorescence images are shown that were taken on a Zeiss Axioimager.Z2 microscope with an Apotome.2 structured illumination accessory using a Plan-Apochromat 40x objective and a cooled CCD camera. The corresponding phase contrast images were taken using a color SLR camera. The identity of each cell is indicated in the phase contrast image (ph: phagocyte; rs: red spherule cell; v: vibratile cell). Live vibratile cells were not bound by any of the lectins under these conditions, and a phase contrast image of one such cell is shown for comparison (**E**).

Phagocytic cells showed a relatively uniform staining with sWGA over the spread out cell body and a more intense signal around the nucleus (Fig 1A). This is consistent with binding to cell surface proteins as the cells are flat distal to and thicker proximal to the nucleus. Red spherule cells are much smaller and globular in shape and but show a similar pattern using sWGA, SJA, STL, and LEL (Fig 1C, 1D, 1F and 1H). In contrast, vibratile cells show a punctate staining with sWGA, STL, and LEL that is restricted to what appear to be large vesicles in the cytosol of the cells (Fig 1B, 1E and 1G). Furthermore, in vibratile cells from some but not all sea urchin tested, PSA shows a fine punctate pattern in the cytoplasm (S1A Fig). It is important to note, that although the fixation procedure applied maintains the phagocyte, red spherule cell, and colorless spherule cell morphologies, the visual appearance of vibratile cells changes dramatically with both their diameter and granularity increasing (compare e.g. Fig 1B to Fig 3E). This suggests that the plasma membrane of vibratile cells might be at least partially permeabilized, and hence the lectins gain access to intracellular structures under these conditions. Employing a higher concentration of aldehyde crosslinking agents maintains the morphology of the cells, but no lectin binding was observed for any of the coelomocytes under these conditions (data not shown) indicating that the corresponding glycan epitopes on the cells were destroyed. Importantly, sWGA and STL stain all red spherule cells (Fig 2). In all other cases, only a fraction of each cell type is bound by each lectin (Fig 2) indicating that subpopulations with distinct molecular features exist. Three subpopulations of phagocytes that differ to a varying degree in their morphology have been described [16] but we could not discern a clear difference in their lectin binding patterns.

The staining results reported above were consistent between coelomocytes from at least three different sea urchins for each lectin, with the exception of PSA and LCA for vibratile cells and PSA for phagocytic cells (Fig 2 and S1 Fig). Rhodamine-coupled PSA and LCA clearly bound to respective fixed cells from one sea urchin but not from others (S1 Fig). The corresponding inter-individual differences in terms of glycosylation are likely biologically relevant and possibly linked to their immune status, and they will therefore be the focus of future studies.

### Fluorescence microscopy of live coelomocytes

The fixation of cells involves the cross-linking of proteins by aldehydes and this chemical modification may alter the ability of the lectins to recognize and bind to respective glycosylated cell surface molecules [19,20]. To determine whether our initial approach using fixed cells missed any glycosylation pattern that can be used to distinguish different live *S*. *purpuratus* coelomocytes, respective stains were performed for all fifteen fluorescently labelled lectins (Fig 3). This is also relevant for potential purification strategies (see below) as those would desirably be executed on live cell samples. Total coelomocyte samples were handled as described above with the exception of the fixation step being omitted and coverslips not being sealed prior to microscopy. Seven out of the fifteen lectins tested showed a readily observable fluorescence signal on some coelomocytes compared to unstained samples, and the signals were consistent between cells from at least two individual sea urchins: sWGA, LCA, PSA, STL, LEL, DSL, and VVL (Fig 3 and S2D and S2E Fig). The remaining eight lectins (GSL-1, GSL-2, PHA-L, PHA-E, SJA, PNA, ECL, Jacalin) did not appear to bind to any of at least 500 individual coelomocytes being screened. Note that all live cells could readily be classified based on their morphology, and hence no gradient purification was required. Interestingly, none of the lectins showed exactly identical staining patterns comparing both approaches (compare Figs 1 and 3, and summarized in Fig 2). The most striking difference is the absence of robust lectin binding to live vibratile cells, consistent with the observation that seemingly only intracellular compartments but not their surface was bound by sWGA, LCA, PSA, STL, and LEL in fixed samples. In addition, some lectins show identical staining for fixed and live cells only for one cell type but not for other coelomocytes: sWGA for phagocytic cells, and STL for red spherule cells, respectively. Other lectins, however, labelled either only live cells (LCA, LEL, and DSL for phagocytic cells, and VVL for red spherule cells) or only fixed cells (sWGA, PSA, STL, and LEL for vibratile cells, and SJA for red spherule cells). Lastly, there are lectins for which the proportion of cells stained varied between live and fixed conditions (sWGA and LEL for red spherule cells). The molecular basis of the observed differences is unclear, but it is conceivable that the fixation with paraformaldehyde altered the accessibility of the corresponding glycan epitopes. Nevertheless assuming that differences in both glycosylation patterns themselves and their accessibility are reflective of different cellular states, their potential use to distinguish and characterize coelomocyte populations remains still valid.

**Fig 4:**
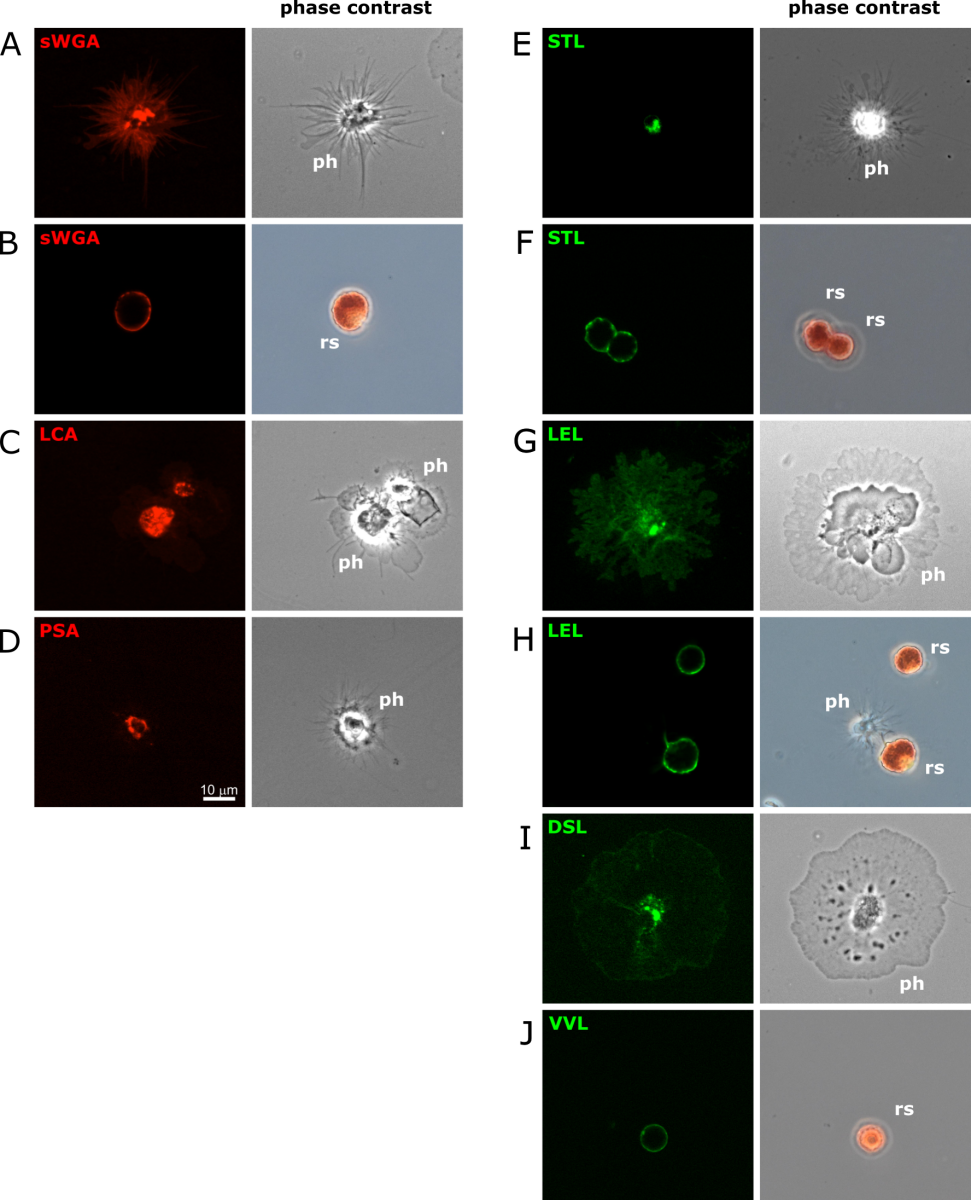
Lectin staining of live coelomocytes. Total coelomocytes were settled onto glass slides, and subsequently stained with the indicated fluorescently labeled lectins (**A**-**D**: rhodamine; **E**-**J**: fluorescein). Representative fluorescence images are shown that were taken on a Zeiss Axioimager.Z2 microscope with an Apotome.2 structured illumination accessory using a Plan-Apochromat 40x objective and a cooled CCD camera. The corresponding phase contrast images were taken using a color SLR camera. The identity of each cell is indicated in the phase contrast image (ph: phagocyte; rs: red spherule cell).

Phagocytic cells change their morphology upon settling down on the surface of glass slides, as they stretch out over a relative large surface. This likely reflects different properties of such cells floating in suspension in the coelomic cavity, compared to those settling down onto and entering into solid tissue. Hence we tested whether the glycosylation pattern of phagocytic cells would differ between cells that were stained in suspension as compared to those that first settled down on glass slides (Figs 2 and 4 and S2F and S2G Fig). Importantly, all staining profiles remained identical with the exception of STL. This lectin appears to bind to phagocytic cells in solution but fails to do so after settling them onto slides. It is plausible that this reflects a difference in the glycosylation state depending on intimate contact with other cells, or that the respective glycan epitopes are inaccessible after settling down. Overall, fluorescent labeled lectin can selectively bind to live coelomocytes. This further supports our idea that the glycosylation patterns forming the basis of this distinction are robust molecular markers to distinguish between different types of coelomocytes and their subpopulations.

### Flow cytometry analysis of live coelomocytes

**Fig 5:**
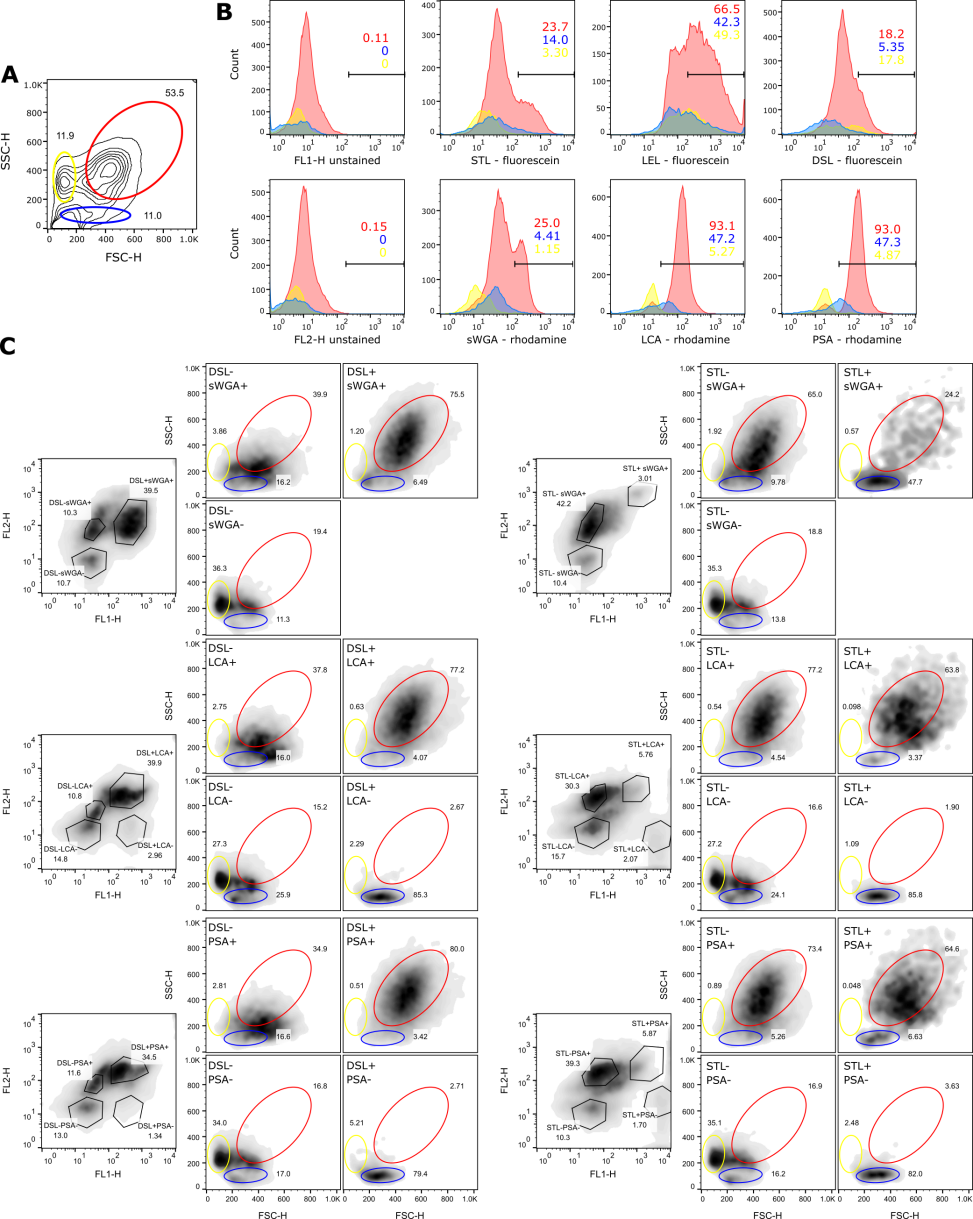
Flow cytometry analysis of lectin stained coelomocytes. (**A**) Forward/side scatter profile of total coelomocytes from sea urchin B. The gating of distinct populations is shown as red, blue, and yellow ovals, and the percentage of live cells in each of these gates is provided. (**B**) Histogram plots of coelomocytes that were either unstained or stained with the indicated fluorescently labelled lectins from sea urchin B. The data from each of the three gates (red, blue, and yellow) is shown as an overlay and the percentage of positive cells is provided. (**C**) Total coelomocytes from sea urchin A were stained with the indicated combinations of fluorescently labeled lectins, and analyzed by flow cytometry. The forward/side scatter profiles of each gated population are show. The gates of the three distinct populations (red, blue, and green ovals) from Fig 5A and the percentage of events in each of the gates are provided.

While the observation of the fluorescence labels of individual cells by microscopy provided insight into the glycosylation patterns of surface molecules on different coelomocyte types, this technique is too “low-throughput” to accurately assess the observed patterns at a population level. Therefore flow cytometry was utilized to analyze the staining profiles of live *S*. *purpuratus* coelomocytes with sWGA, LCA, PSA, STL, LEL, and DSL. The non-fluorescent properties of cells that are routinely assessed by flow cytometers, forward scatter (FSC–correlated with cell size) and side scatter (SSC–correlated with cell granularity), revealed a broad distribution within the entire coelomocyte set (Fig 5A). The largest population of cells (referred “red”) that could be readily identified in all individuals tested (at least 60% of all live cells) likely corresponds to the phagocytic cells, as they represent the dominant coelomocyte type in the coelomic fluid [14]. Other populations (referred to as “yellow” and “blue”, respectively) are clearly discernible (Fig 5A) but the relative numbers of cells in these populations varies between sea urchins (data not shown) in line with differences in red spherule cell, colorless spherule cell, and vibratile cell numbers. A preliminary assignment of cell types to the respective populations in the FSC/SSC profile will be discussed below.

The histogram plots of cells stained with one fluorescently labelled lectin, revealed clear differences in the staining of individual coelomocytes (Fig 5B) as was expected from the microscopy data. In almost all cases the “negative” population shows a stronger fluorescence signal than the cells that were unstained. This is likely due to low affinity binding to glycosylation structures that are not the cognate targets of the respective lectins. To test the specificity of the lectin staining, chitin hydrolysate (sWGA, LEL, STL, and DSL), α-methylmannoside (LCA and PSA), and N-acetylgalactosamine (VVL and SJA) were used as competitors (S4 Fig). Lectin binding was inhibited to varying degrees by their respective competitors at their recommended concentration (S4A Fig) which likely reflects the fact that these small molecules have a lower affinity than the cognate targets, polysaccharides on the surface of cells. Importantly, the small molecules were selective (S4B Fig). However, even in the presence of the respective blocking agents, the coelomocytes show a stronger fluorescence than the unstained cells as discussed above.

Importantly, the distinct cell populations defined by FSC/SSC (Fig 5A), differ in their fluorescence signals (Fig 5B). For example almost all phagocytic cells are stained with sWGA, but they clearly fall into two populations: sWGA^+^ and sWGA^hi^ (Fig 5A and 5B–red). In contrast, the blue and yellow populations are largely sWGA^+^ and sWGA^-^, respectively. This data indicates that the binding of fluorescently-labeled lectins results in a wide range of intensities on different coelomocytes that can be readily distinguished by flow cytometry.

To test whether some lectins recognize identical, distinct, or partially overlapping populations of coelomocytes, double staining experiments were conducted with combinations of the fluorescein- and rhodamine-coupled lectins sWGA, LCA, PSA, DSL, STL, PSA, and LEL (Fig 5C and S1 Fig). As the LEL signals remained too bright despite reducing the amount of lectin used for staining we decided to not discuss the data in the following section. Most combinations of lectins stained three or four clearly distinct populations of double-negative, single-positive, and double positive cells indicating that the glycosylation patterns are partially overlapping. Using back-gating we determined the FSC/SSC profiles of cells with distinct fluorescence profiles (Fig 5C) and attempted to correlate them to the FSC/SSC profiles described above (Fig 5A–“red”, “yellow”, and “blue”). As expected, the largest populations (DSL^+^sWGA^+^, DSL^+^LCA^+^, DSL^+^PSA^+^, STL^-^sWGA^+^, STL^-^LCA^+^, and STL^-^PSA^+^) overlap with the largest population discernible in the FSC/SSC plot (“red”). This is in agreement with both, the fluorescence microscopy data (Fig 4) and the previous assignment as phagocytic cells based on them being the most abundant cells in the coelomic fluid. Interestingly, however, some DSL^+^sWGA^+^ and STL^-^sWGA^+^ cells fall within the range of the “blue” FSC/SSC profile. The double negative cells (DSL^-^sWGA^-^, DSL^-^LCA^-^, DSL^-^PSA^+^, STL^-^sWGA^-^, STL^-^LCA^-^, and STL^-^PSA^-^) do largely overlap with the “yellow” FSC/SSC population and some of them (DSL^-^LCA^-^, DSL^-^PSA^-^, STL^-^LCA^-^, STL^-^PSA^-^) do also contribute to a varying extend with a “blue” FSC/SSC profile. Lastly, DSL^+^LCA^-^, DSL^+^PSA^-^, STL^+^sWGA^+^, STL^+^LCA^-^, and STL^+^PSA^-^ coelomocytes are unique to the “blue” FSC/SSC population. Combined with the absence of any lectin staining for vibratile cells in the microscopy experiments, we conclude that the “yellow” population and the majority of the double negative cells represent the vibratile cells. Thus the “blue” population likely contains the majority of red spherule cells, and the observation that these cells can be STL^+^ or sWGA^+^, but LCA^-^ and PSA^-^ (Fig 2) is largely consistent with this notion. The simultaneous presence of DSL^+^ cells could be explained by a subpopulation of phagocytic cells with a similar FSC/SSC profile, or by technological challenges that preclude the detection of DSL^+^ red spherule cells in the microscopy studies. Importantly, the properties of colorless spherule cells remained elusive in these experiments, and they are likely included in one of the populations described above.

**Fig 6:**
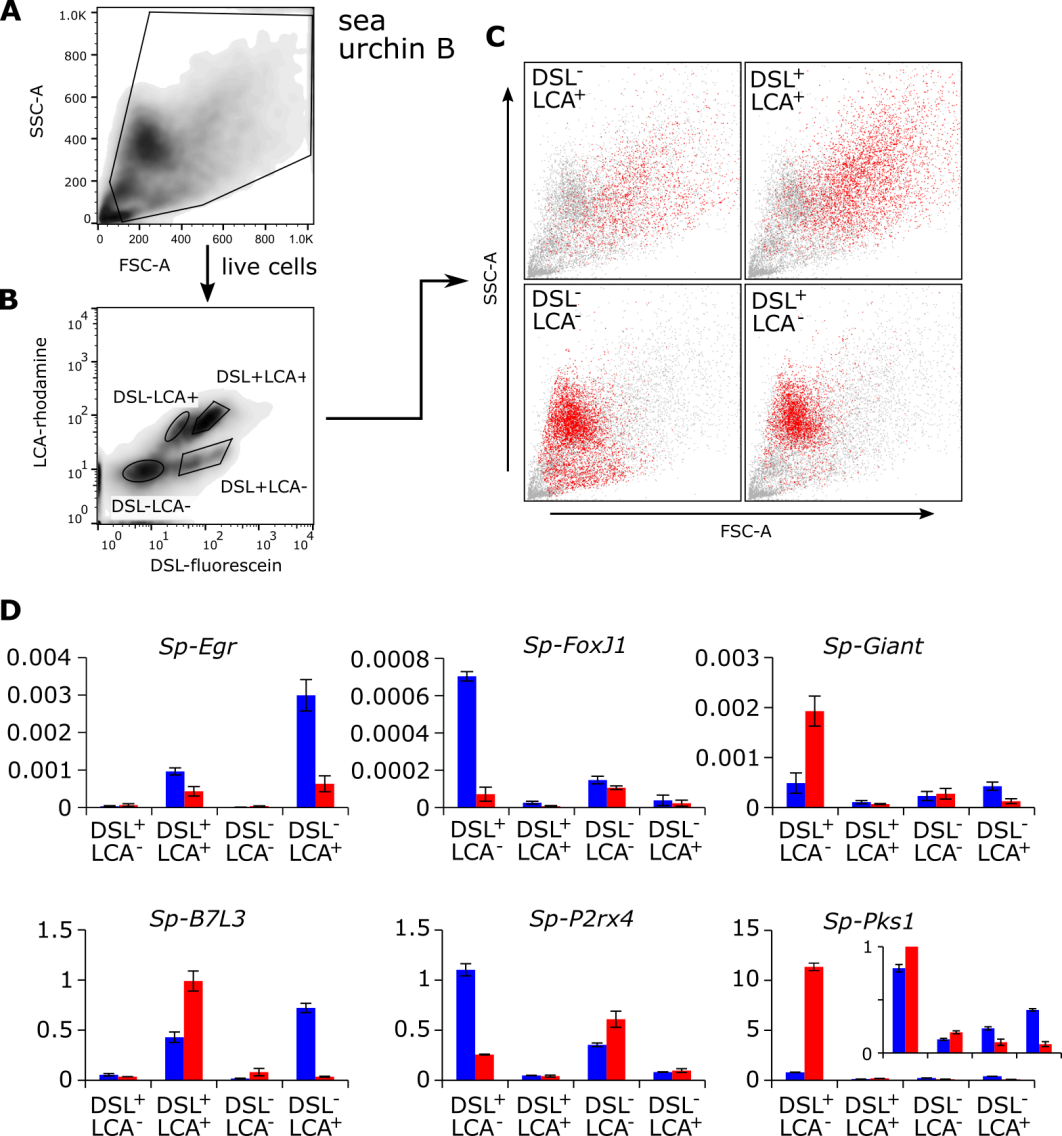
Flow cytometry based cell sorting of lectin-labeled coelomocytes. Total coelomocytes from sea urchin B were stained with DSL-fluorescein and LCA-rhodamine. Live cells (**A**) were gated based on their forward/side scatter profile, and four different populations (**B**) were sorted based on their distinct fluorescence profiles. (**C**) The forward/side scatter profiles of each indicated population (red dots) was overlaid on that of all cells in the sample (gray dots). (**D**) The expression levels of the phagocyte specific genes *Sp-B7L3* and *Sp-Egr*, the genes unique for a mix of vibratile and colorless spherule cells *Sp-P2rx4* and *Sp-FoxJ1*, and the red spherule cell specific genes *Sp-Pks1* and *Sp-Giant* were quantified in each of the indicated sorted cell populations from sea urchin B (blue) and sea urchin C (red) by qRT-PCR. Values are normalized to the levels of *Sp-RPL39* in each sample. The means of two technical replicates are shown and the error bars indicate the standard error of the mean.

Based on the double-staining data (Fig 5C), the red spherule cells can likely be divided into STL^-^ and STL^+^ as well as DSL^-^ and DSL^+^ subpopulations. The same applies to phagocytic cells. This suggests that STL and DSL might bind to common glycosylation patterns linked to activation or potentially apoptosis in both cell types. Importantly, whether DSL^+^ and STL^+^ populations are identical or non-overlapping requires further experimental testing. Lastly, the double staining patterns are largely consistent between different sea urchins (data not shown), but there was at least one notable exception: the DSL^+^LCA^-^ population in sea urchin A (Fig 5C) seems to largely consist of red spherule cells, but in the sea urchins B and C used for the cell sorting experiment described below, DSL^+^LCA^-^ includes both, vibratile cells and red spherule cells (Fig 6C and S5 and S6 Figs). The basis of these differences remains elusive, but may be reflective of DSL binding to a glycosylation features that are markers of common states that can be acquired by all coelomocytes.

### Purification of coelomocytes based on lectin staining patterns

To test whether fluorescence based cell sorting can be used to purify coelomocyte populations based on their lectin binding properties, fluorescein-coupled DSL and rhodamine-coupled LCA were employed to stain the total pool of coelomocytes from two individual sea urchins (named B and C, respectively). This combination showed four distinct populations in our analytical flow cytometry, and now these four different populations (DSL^-^LCA^-^, DSL^+^LCA^-^, DSL^-^LCA^+^, and DSL^+^LCA^+^) were separated by flow cytometry using appropriate gates (Fig 6A and 6B and S6 Fig). The use of PBS instead of CMFSW-EI as the sheath fluid (a limitation based on regulations of our flow cytometry facility) precluded us from reanalyzing and visually observing cells by flow cytometry of microscopy after sorting. Thus we decided to determine the cell type(s) contained in each population based on the expression of signature genes that we identified in a recent RNAseq experiment of density gradient-purified coelomocytes (SDF, unpublished data). qPCR was used to test the expression of six genes: SPU_020457 (*Sp-B7L3*) and SPU_015358 (*Sp-Egr*) (phagocyte-specific), SPU_002895 (*Sp-PKS1*) and SPU_014528 (*Sp-Giant*) (red spherule cell-specific), and SPU_019489 (*Sp-P2rx4*) and SPU_027969 (*Sp-FoxJ1*) (specific for a mixed population of vibratile and colorless spherule cells). To account for the differences in the numbers of cells collected, all expression values were normalized to that of *Sp-Rpl39* for each population. The genes that are preferentially expressed in the DSL^+^LCA^+^ population are the phagocyte markers *Sp-B7L3* and *Sp-Egr* consistent with our earlier assignment of this population to be phagocytes based on fluorescence microscopy and analytical flow cytometry data (Figs 4 and 5). The DSL^-^LCA^+^ cell show a very similar gene expression profile population but at least in sea urchin C low levels of *Sp-PKS1* and *Sp-Giant* transcripts are also readily detectable indicating the presence of red spherule cells (Fig 6C). In both sea urchins, the sorted DSL^-^LCA^-^ population is characterized by the expression of the vibratile and colorless spherule cell population markers *Sp-P2rx4* and *Sp-FoxJ1*, but in sea urchin B these cells also express red spherule cell markers. Lastly, in DSL^+^LCA^-^ cells markers for red spherule cells and vibratile/colorless spherule cells were both clearly expressed, although their levels differed between the two individuals. This in part could reflect the difference in the ratio of these cell types between the two sea urchins. Importantly, overall the gene expression pattern (derived from density gradient purified cell fractions) correlates well with the lectin staining patterns observed in the microscopy and analytical flow cytometry experiments described above. In summary, these results indicate that fluorescently labeled lectins can be used purify different coelomocyte types and subpopulations thereof by flow cytometry, and corresponding RNA preparations are suitable to establish the gene expression profiles of these cells.

## Discussion

The four main coelomocytes within *S*. *purpuratus* and other echinoderms are defined by their distinct morphologies which reflect their distinct (albeit currently largely unknown) biological functions. Each cell type exhibited defined lectin binding patterns which corresponds to distinct glycosylation patterns of their cell surface proteins, and this in turn is controlled by glycosyltransferase activities that are linked to the biological function and state of each cell. The precise glycosylation features recognized by each lectin are only beginning to be accurately defined [21]. Hence it is unclear which glycosyltransferases are critical to allow for the binding of a specific lectin to the surface of such cell. A transcriptome analysis from respective purified populations has the potential to reveal their identity. We have already generated RNAseq data for different cell fractions based on a density gradient purification method, but no distinct sets of glycosyltransferases emerged from an initial analysis that would allow predicting post-translational modifications unique to each coelomocyte type. The molecular composition of glycans on a mixed population of coelomocytes of *Paracentrotus lividus*, a sea urchin, was recently analyzed by mass spectroscopy, and they confirmed the presence of sialic acid on these cells using fluorescently-labelled sWGA [22]. Our results now confirm this observation in a different echinoderm species, and extend it by analyzing distinct coelomocyte types. Interestingly, immune cell type specific lectin binding patterns are not unique to sea urchin but have also been reported in earthworms where WGA strongly binds to amoebocytes but not to eleocytes [23].

Some of the lectins were found to bind to all cells of a given type (e.g. STL to all red spherule cells) in all individual sea urchin tested, and hence the respective glycosylation features are important for the function of all cells of this type. Other lectins (e.g. PSA), however, differed in their staining pattern within or between individuals (Fig 2). They either only bound to some cells of a given cell type, or only did so in one but not another sea urchin. These findings likely reflect distinct subpopulations within each coelomocyte type. Based on their size and their actin cytoskeleton, phagocytes can be assigned to three subgroups, discoidal cells, polygonal cells, and small phagocytes [16], and it is plausible that these subgroups account for differences in lectin binding. Our initial experiments, however, did not reveal a clear correlation between these subgroups and the observed glycosylation patterns, but this could be due our assignment to each category based on their morphology in the phase contrast image. Combined staining with anti-actin antibodies to visualize the cytoskeleton and fluorescently labeled lectins could potentially reveal such correlations. Similarly, in *S*. *purpuratus* larvae two morphologically identical pigment cell subtypes that differ in their gene expression profiles have been described [24], and it is possible that the red spherule cells of adult sea urchin share similar features that could reflected in their ability to be bound by different lectins. Furthermore, differences in glycosylation patterns could also correspond to different activation states or stages of development of a given cell type. Although we attempted to study resting coelomocytes in the absence of any infection or inflammation, it is possible that the simple act of retrieving the cells from the sea urchin was sufficient to activate at least some of the cells. Importantly, there is precedence for changes in glycosylation patterns upon immune cell activation, namely ability of PNA to label activated mouse B lymphocytes [10]. The presence of some DSL^+^ and STL^+^ cells for at least two coelomocyte types in the flow cytometry experiment could reflect such a common activation marker. In addition, genetic differences between each sea urchin, and differences in their disease history and current immune status could also contribute to differences in lectin binding profiles. Performing lectin staining studies on cells from a large number of individual sea urchins including infection experiments will help to distinguish between shared constitutive glycosylation patterns defining cellular identify, and those that are inducible. As molecular features and markers that correlate to activation states, developmental stages, and coelomocyte subtypes emerge from future studies of echinoderm immunity, these currently seemingly non-consistent staining patterns will likely be resolved.

While we were not able to define any lectin binding profile for colorless spherule cells due to their low abundance in the coelomic cavity of adult *S*. *purpuratus*, the vibratile cells were unique in their inability of being recognized in their native state by any of the fifteen lectins used in this study (with DSL being a potential exception), while fixed vibratile cells revealed the presence of diverse glycosylation patterns in intracellular compartments. It is tempting to speculate that these are linked to proteins that will be secreted from those cells upon activation, and a role of these cells in clotting consistent with such secretory processes has been discussed in the literature [25].

The flow cytometry experiments strongly suggest that an appropriate combination of FSC/SSC gating combined with appropriate fluorescently labeled lectins should enable us to purify homogenous coelomocyte population for subsequent functional studies and the assessment of their gene expression profiles. The FSC/SSC profiles are relatively coarse measures of cell morphology that are overlapping, and this is likely reflected in the very large but not complete overlap of cell populations defined by these physical features and the molecular properties of lectin binding and gene expression. An iterative process in which increased cell purities will lead to the identification of better molecular markers that will in turn could be used to obtain higher cell purities will resolve some of the apparent inconsistencies observed in our study. One important next step will be using lectin-based cell purification to identify candidate genes that encode cell surface markers for exclusive to unique coelomocyte populations, and to subsequently develop appropriate monoclonal antibody reagents. Overall, we predict that the study presented here will pave the way towards using combination of lectins and antibodies to facilitate the dissection of the *S*. *purpuratus* immune cell populations beyond the level of their morphologies. This will set the stage for detailed studies of their properties, development and function during immune responses that are currently impossible to conduct. Importantly, a recent flow cytometric analyses of earthworm coelomocytes included lectins to distinguish cell populations [23], highlighting the applicability of our approach for the analysis of invertebrate immune cell populations in general.

## Supporting information

S1 Fig Unique lectin staining pattern of fixed coelomocytes from individual sea urchins. The lectin staining patterns shown here were not consistent among coelomocytes from all individuals tested as each was only observed in one sea urchin but not in at least two other individuals. (**A+B**) Total coelomocytes were directly settled on glass slides, or (**C**) first separated over a density gradient to obtain cell fractions enriched for phagocytes (ph), vibratile cells (v), and red spherule cells (rs). Cells were fixed with paraformaldehyde, and stained with DAPI and the indicated lectins that were labeled with rhodamine. Representative images were taken on a Zeiss Axioimager.Z2 microscope with a cooled CCD camera using (**A+B**) a Plan-Apochromat 40x objective, or (**C**) an Apotome.2 structured illumination accessory and a Plan-Apochromat 40x objective. Respective phase contrast images were taken (without the Apotome.2 feature) to confirm the identity of each cell. The images for the fluorescent channels are shown individually and merged.
(TIF)

S2 Fig Unstained coelomocytes. (**A-C**) Density gradient purified coelomocytes (ph: phagocytes, v: vibratile cells, and rs: red spherule cells) were settled and glass slides, fixed with paraformaldehyde, and stained with DAPI. (**D-G**) Total live coelomocytes were settled or added to glass slides and handled according to Fig 3 with no lectin-dye conjugates added. Representative images in the Rhodamine, FITC, and DAPI channels were taken on a Zeiss Axioimager.Z2 microscope with a cooled CCD camera using an Apotome.2 structured illumination accessory and a Plan-Apochromat 40x objective. The exposure times were identical to those used in Fig 1 for stained samples. Respective phase contrast images were taken (without the Apotome.2 feature) to confirm the identity of each cell. The images for the fluorescent channels are shown individually and merged. Note that no pictures were taken in the DAPI channel for live cells and in the FITC channel for phagocytic cells as no fixed phagocyte showed binding to lectin-FITC conjugates (see Fig 1).
(TIF)

S3 Fig Competition assay of lectin staining of fixed coelomocytes. Total coelomocytes were separated over a density gradient to obtain cell fractions enriched for phagocytes (ph), vibratile cells (v), and red spherule cells (rs). Cells were settled on glass slides, fixed with paraformaldehyde, and stained with DAPI and the indicated lectins that were labeled with (**A**-**D**) rhodamine or (**E-H**) fluorescein in the presence of chitin hydrolysate (ch) or N-acetylgalactosamine (N-ag). Representative images were taken on a Zeiss Axioimager.Z2 microscope with an Apotome.2 structured illumination accessory using a Plan-Apochromat 40x objective and a cooled CCD camera. The exposure times were identical to those used for the respective stained coelomocytes in Fig 1. Respective phase contrast images were taken (without the Apotome.2 feature) to confirm the identity of each cell. The images for the fluorescent channels are shown individually and merged.
(TIF)

S4 Fig Lectin binding competition assay of coelomocytes. (A) Histogram plots of live coelomocytes that were either unstained (red), stained with the indicated fluorescently labelled lectins (blue), or stained with the indicated fluorescently labelled lectin in the presence of the indicated competitors (green)(ch: chitin hydrolysate, α-methylmannoside, or N-ag: N-acetylgalactosamide). The data from each of the three samples is shown as an overlay. The cells for this dataset were obtained from four individual sea urchins.
(TIF)

S5 Fig Flow cytometry analysis of lectin stained coelomocytes. (**A**) Total coelomocytes from sea urchin A were stained with the indicated combinations of fluorescently labeled lectins, and analyzed by flow cytometry. The forward/side scatter profiles of each gated population are shown and gates corresponding to the distinct populations (shown in Fig 5A) are shown (red, yellow, and blue ovals) including the percentage of cells falling within them. (**B**) Total coelomocytes from sea urchin B were stained with DSL-fluorescein and LCA-rhodamine. The forward/side scatter profiles of each gated population are shown as in (A).
(TIF)

S6 Fig Flow cytometry based cell sorting of lectin-labeled coelomocytes. Total coelomocytes from sea urchin C were stained with DSL-fluorescein and LCA-rhodamine. Live cells (**A**) were gated based on their forward/side scatter profile, and four different populations (**B**) were sorted based on their distinct fluorescence profiles. (**C**) The forward/side scatter profiles of each indicated population (red dots) was overlaid on that of all cells in the sample (gray dots).
(TIF)

S1 Table Gene expression analysis qRT-PCR data Fig 6C in tabular format. (XLSX)
