# Supplementary figures and images for: Lectins identify distinct populations of coelomocytes in Strongylocentrotus purpuratus
Source: PLoS One. 2017 Nov 10;12(11):e0187987. doi: 10.1371/journal.pone.0187987 (PMC5695280; doi:10.1371/journal.pone.0187987)

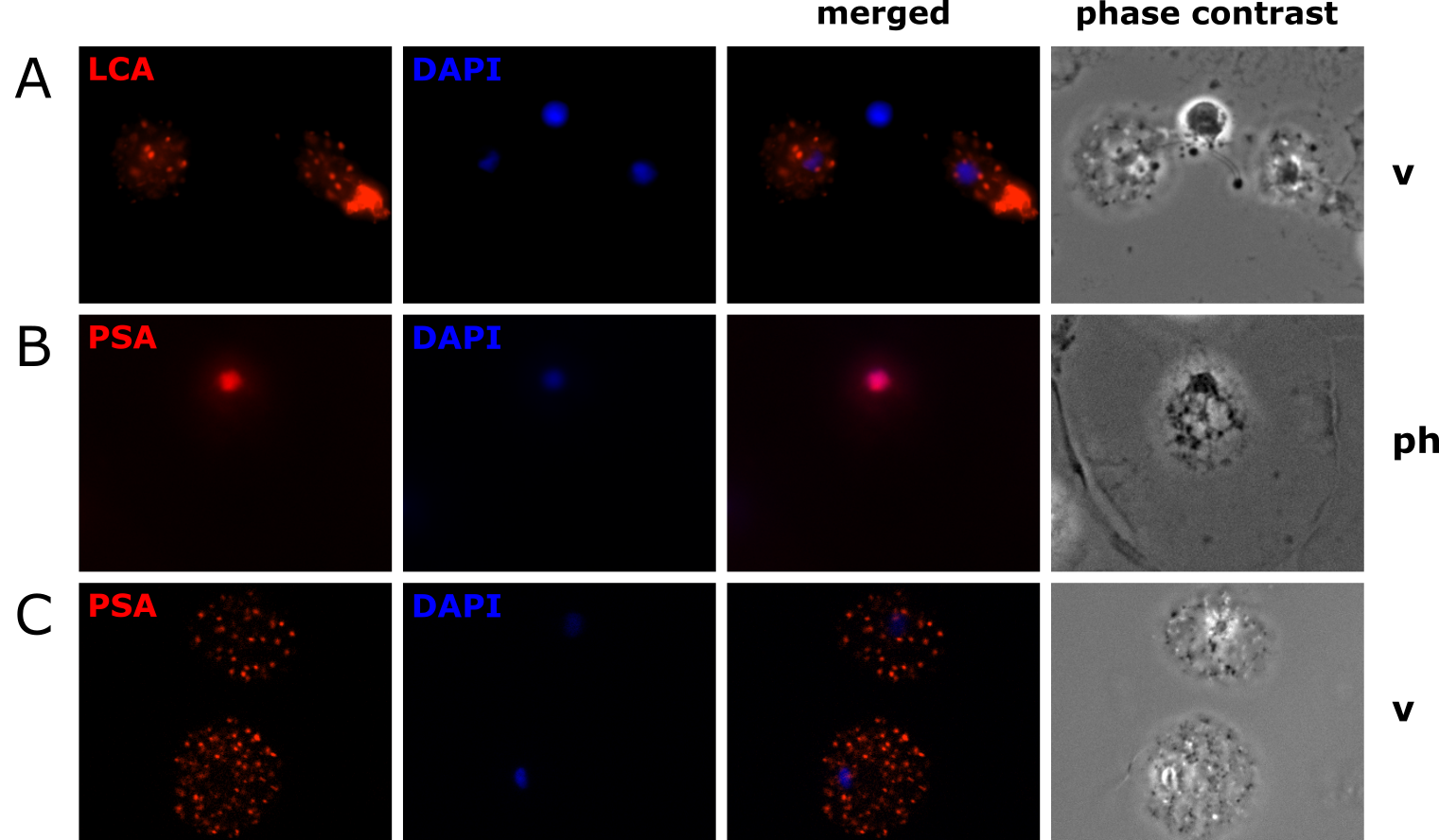

Supplement: S1 Fig — The lectin staining patterns shown here were not consistent among coelomocytes from all individuals tested as each was only observed in one sea urchin but not in at least two other individuals. (A+B) Total coelomocytes were directly settled on glass slides, or (C) first separated over a density gradient to obtain cell fractions enriched for phagocytes (ph), vibratile cells (v), and red spherule cells (rs). Cells were fixed with paraformaldehyde, and stained with DAPI and the indicated lectins that were labeled with rhodamine. Representative images were taken on a Zeiss Axioimager.Z2 microscope with a cooled CCD camera using (A+B) a Plan-Apochromat 40x objective, or (C) an Apotome.2 structured illumination accessory and a Plan-Apochromat 40x objective. Respective phase contrast images were taken (without the Apotome.2 feature) to confirm the identity of each cell. The images for the fluorescent channels are shown individually and merged. (TIF) [file pone.0187987.s001.tif]

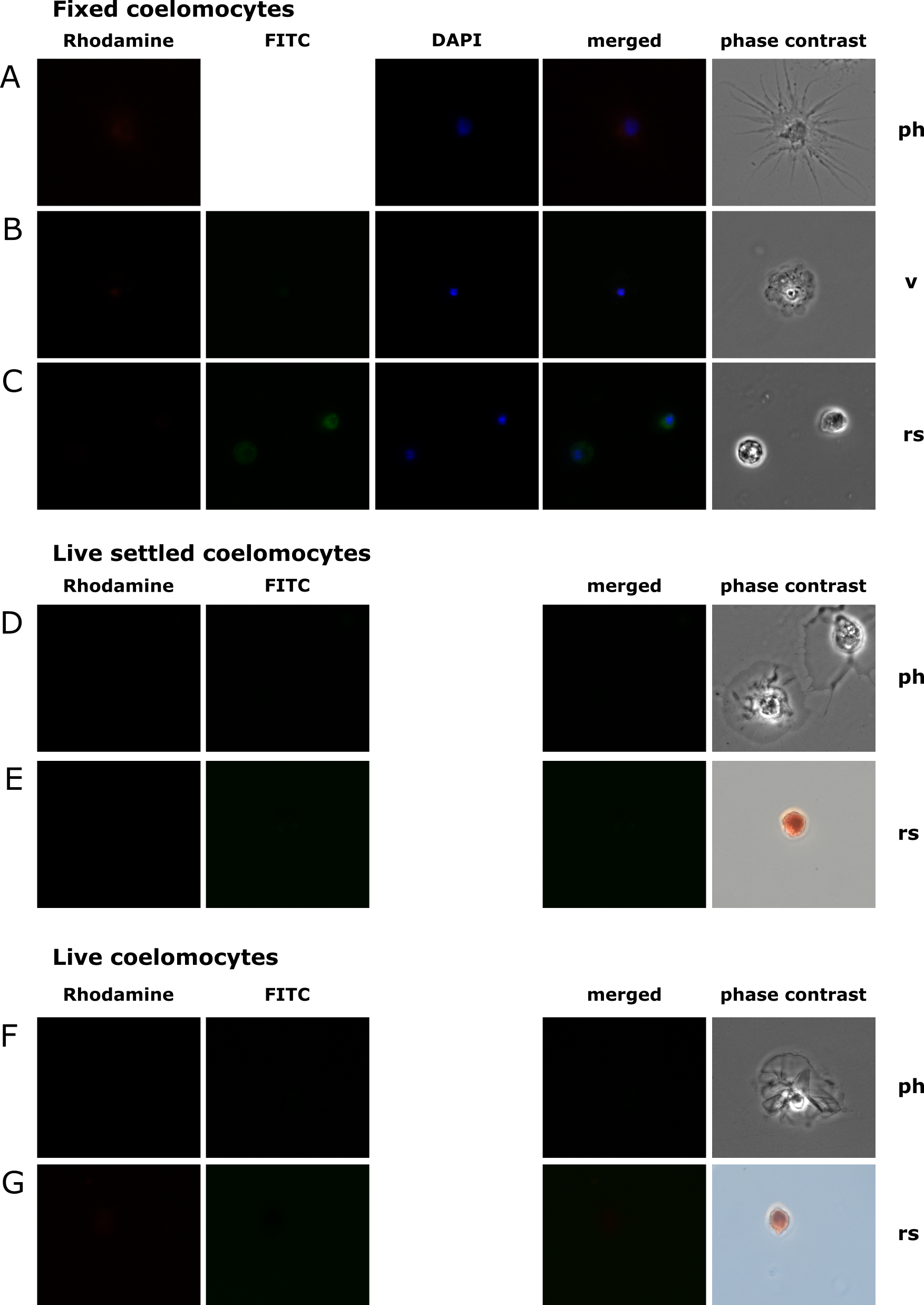

Supplement: S2 Fig — (A-C) Density gradient purified coelomocytes (ph: phagocytes, v: vibratile cells, and rs: red spherule cells) were settled and glass slides, fixed with paraformaldehyde, and stained with DAPI. (D-G) Total live coelomocytes were settled or added to glass slides and handled according to Fig 3 with no lectin-dye conjugates added. Representative images in the Rhodamine, FITC, and DAPI channels were taken on a Zeiss Axioimager.Z2 microscope with a cooled CCD camera using an Apotome.2 structured illumination accessory and a Plan-Apochromat 40x objective. The exposure times were identical to those used in Fig 1 for stained samples. Respective phase contrast images were taken (without the Apotome.2 feature) to confirm the identity of each cell. The images for the fluorescent channels are shown individually and merged. Note that no pictures were taken in the DAPI channel for live cells and in the FITC channel for phagocytic cells as no fixed phagocyte showed binding to lectin-FITC conjugates (see Fig 1). (TIF) [file pone.0187987.s002.tif]

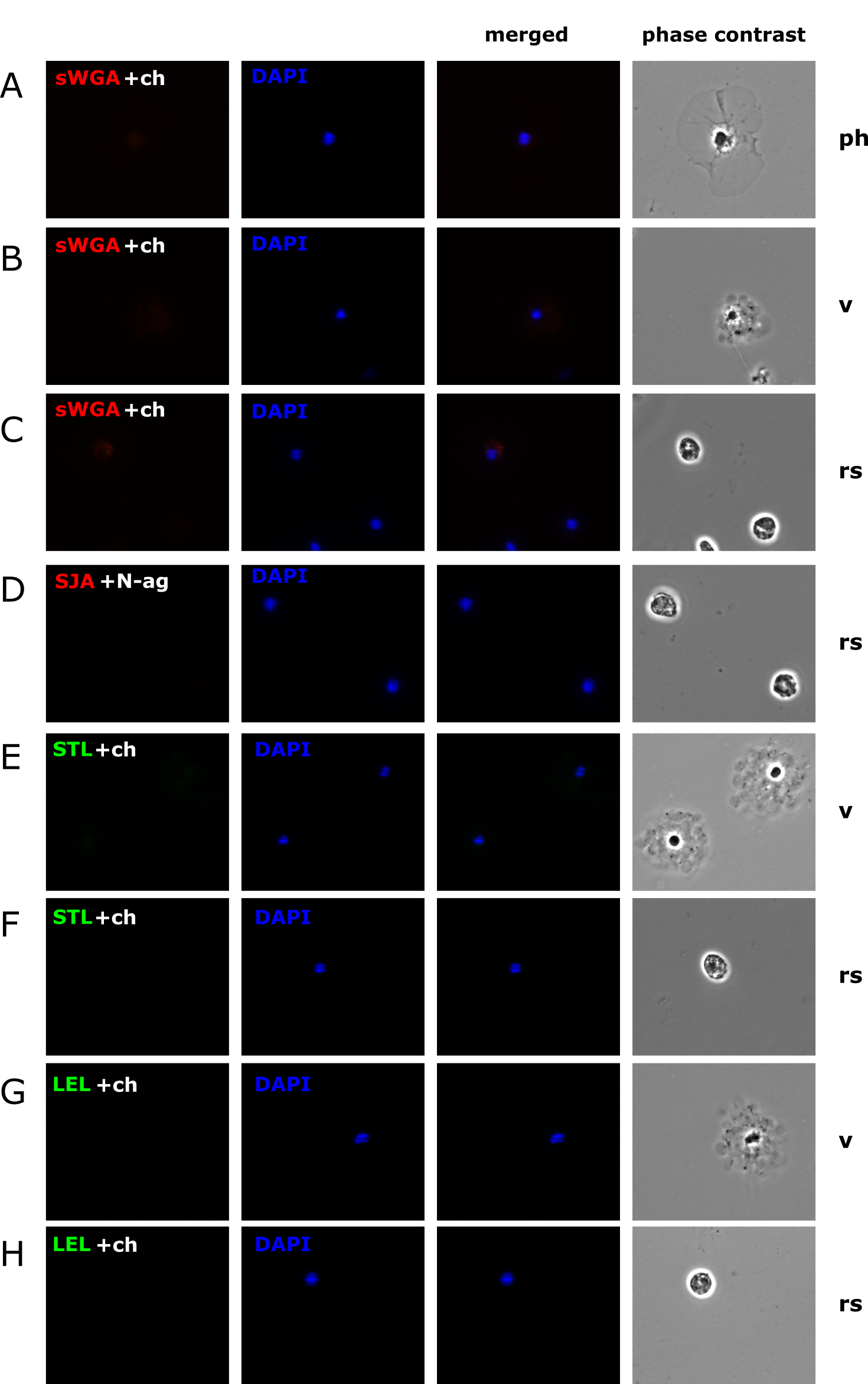

Supplement: S3 Fig — Total coelomocytes were separated over a density gradient to obtain cell fractions enriched for phagocytes (ph), vibratile cells (v), and red spherule cells (rs). Cells were settled on glass slides, fixed with paraformaldehyde, and stained with DAPI and the indicated lectins that were labeled with (A-D) rhodamine or (E-H) fluorescein in the presence of chitin hydrolysate (ch) or N-acetylgalactosamine (N-ag). Representative images were taken on a Zeiss Axioimager.Z2 microscope with an Apotome.2 structured illumination accessory using a Plan-Apochromat 40x objective and a cooled CCD camera. The exposure times were identical to those used for the respective stained coelomocytes in Fig 1. Respective phase contrast images were taken (without the Apotome.2 feature) to confirm the identity of each cell. The images for the fluorescent channels are shown individually and merged. (TIF) [file pone.0187987.s003.tif]

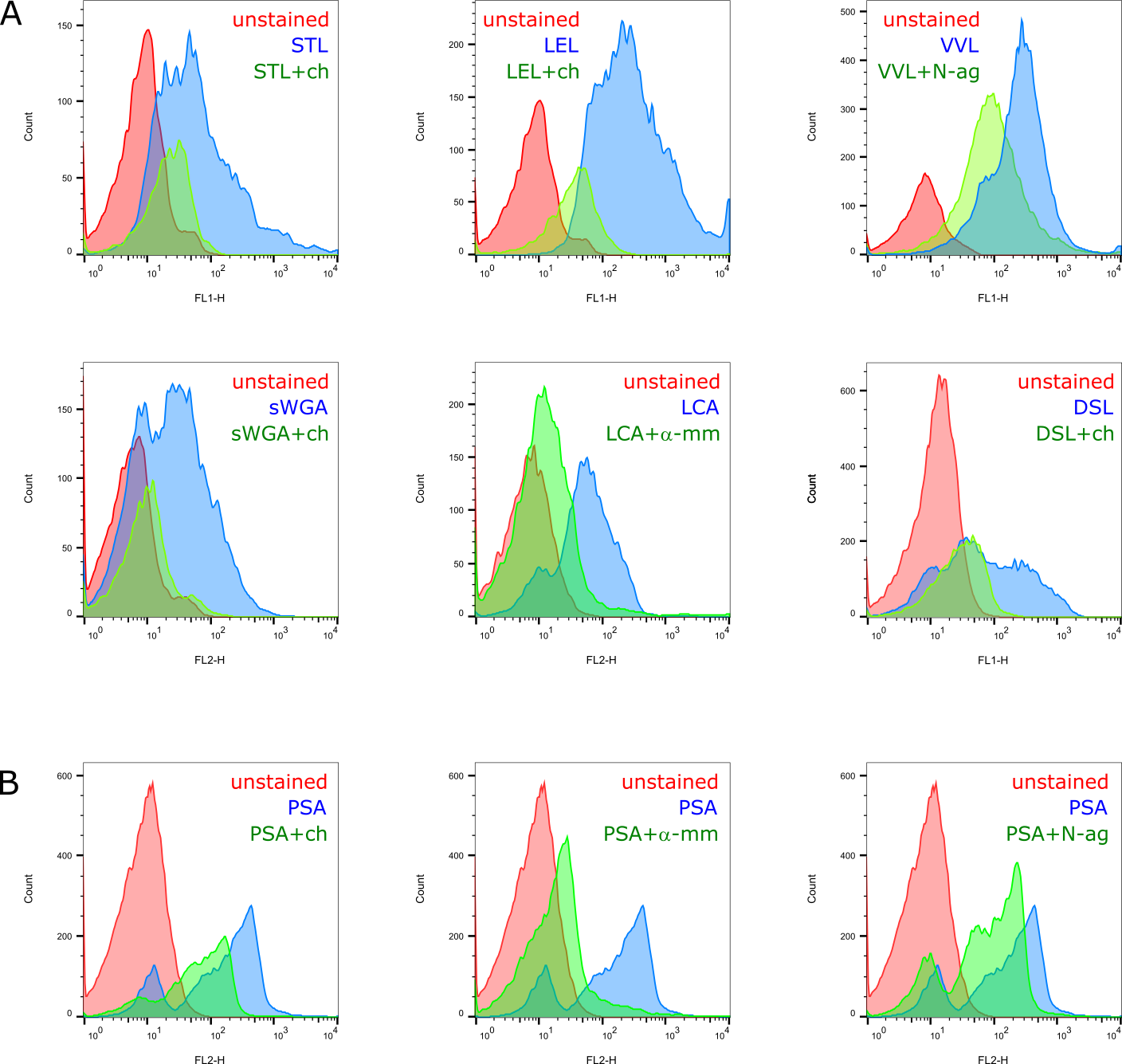

Supplement: S4 Fig — (A) Histogram plots of live coelomocytes that were either unstained (red), stained with the indicated fluorescently labelled lectins (blue), or stained with the indicated fluorescently labelled lectin in the presence of the indicated competitors (green)(ch: chitin hydrolysate, α-methylmannoside, or N-ag: N-acetylgalactosamide). The data from each of the three samples is shown as an overlay. The cells for this dataset were obtained from four individual sea urchins. (TIF) [file pone.0187987.s004.tif]

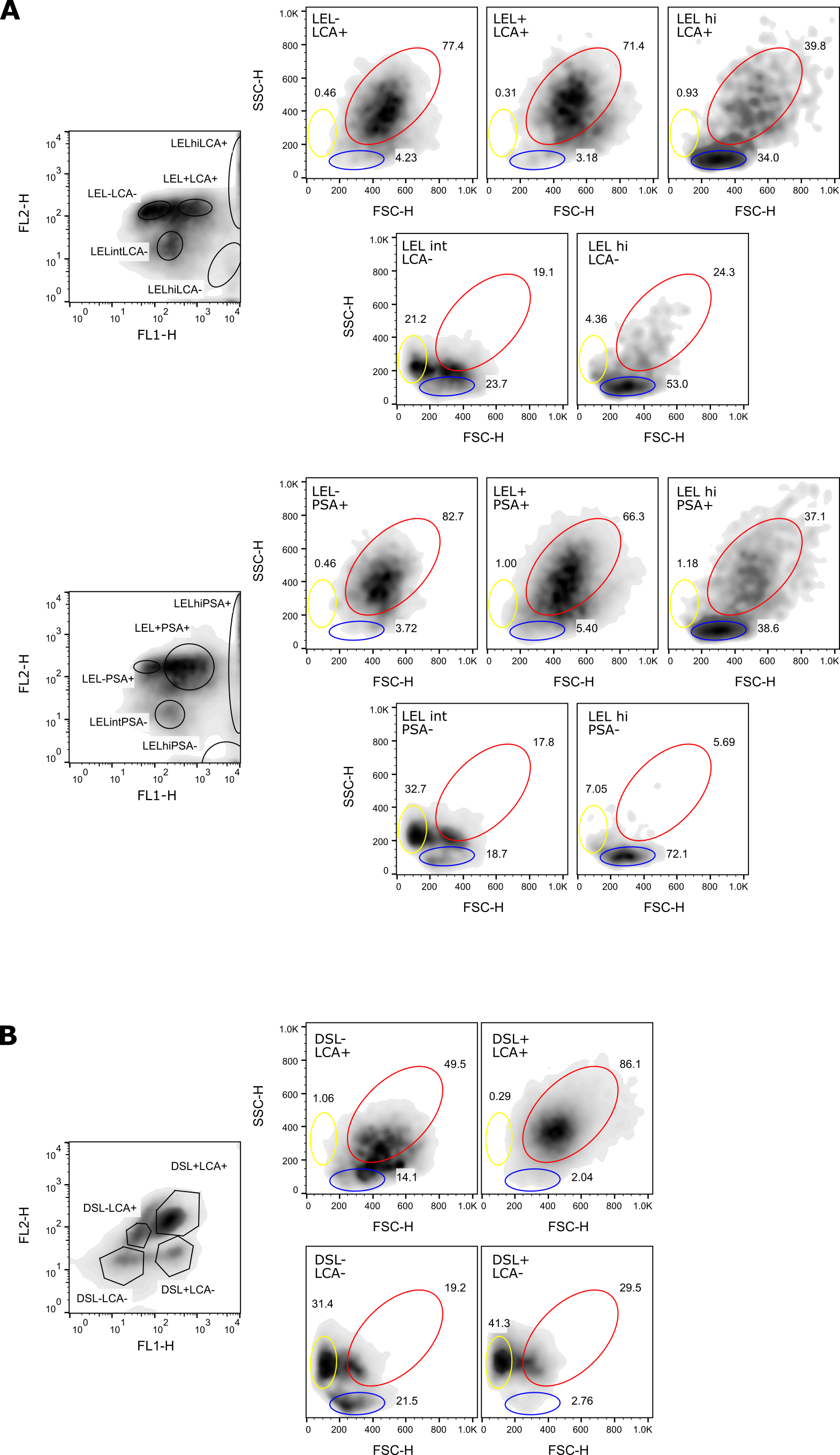

Supplement: S5 Fig — (A) Total coelomocytes from sea urchin A were stained with the indicated combinations of fluorescently labeled lectins, and analyzed by flow cytometry. The forward/side scatter profiles of each gated population are shown and gates corresponding to the distinct populations (shown in Fig 5A) are shown (red, yellow, and blue ovals) including the percentage of cells falling within them. (B) Total coelomocytes from sea urchin B were stained with DSL-fluorescein and LCA-rhodamine. The forward/side scatter profiles of each gated population are shown as in (A). (TIF) [file pone.0187987.s005.tif]

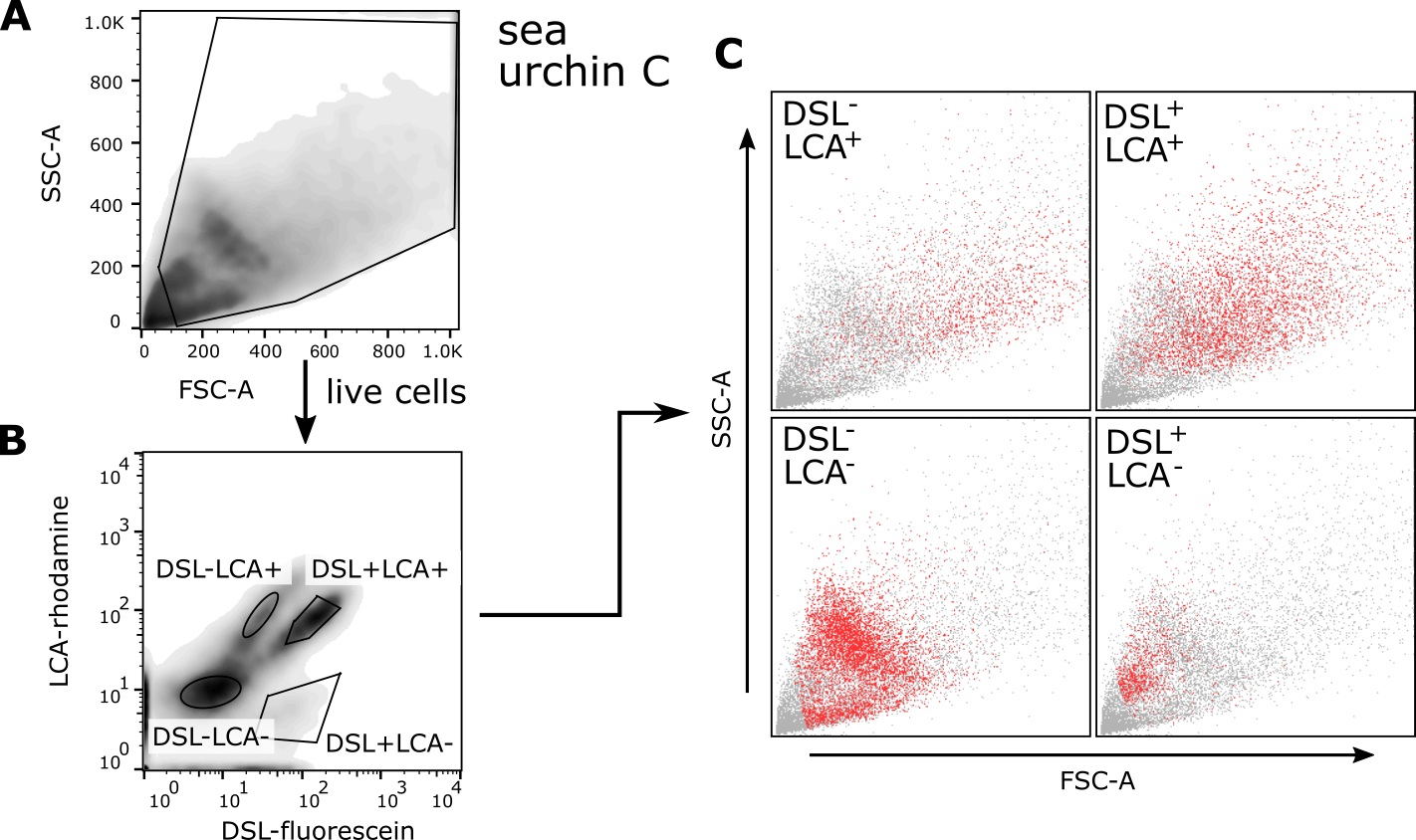

Supplement: S6 Fig — Total coelomocytes from sea urchin C were stained with DSL-fluorescein and LCA-rhodamine. Live cells (A) were gated based on their forward/side scatter profile, and four different populations (B) were sorted based on their distinct fluorescence profiles. (C) The forward/side scatter profiles of each indicated population (red dots) was overlaid on that of all cells in the sample (gray dots). (TIF) [file pone.0187987.s006.tif]
